# *Rickettsia felis* Infection in Man, France

**DOI:** 10.3201/eid1507.090029

**Published:** 2009-07

**Authors:** Aurélie Renvoisé, Antoine-Yves Joliot, Didier Raoult

**Affiliations:** Université de la Méditerranée, Marseille, France (A. Renvoisé, D. Raoult); Centre Hospitalier de Salon de Provence, Salon-de-Provence, France (A.Y. Joliot)

**Keywords:** Keywords: Rickettsia felis, flea-borne spotted fever, Rickettsia, vector-borne infections, France, letter

To the Editor: **In August 2008, a 64-year-old man was admitted to the Salon-de-Provence Hospital, France. He had fever (39°C) and a maculopapular rash. No eschars or adenopathy were noted. The patient had a relatively mild illness; the only abnormal laboratory values were elevated aminotransferase levels (aspartate aminotransferase 85 U/L and alanine aminotransferase 135 U/L). The man was an agricultural worker who had originated from Algeria but at this time lived in a shelter in southern France. His potential for contact with dogs in his environment was noted, but no history of flea exposure was elicted. This disease was postulated to be rickettsiosis because no other cause for his fever and rash was evident. Doxycycline was then administered, and the patient rapidly improved.**

**Serum testing at the Unité des Rickettsies (Marseilles, France), using a multiple-antigen immunofluorescent assay (*1*), showed the following titers: spotted fever group (SFG) (e.g., *Rickettsia felis*, *R. conorii*, *R. aeschlimannii*, *R. massiliae*) 1,024 and 512 for immunoglobulin (Ig) G and IgM, respectively, and typhus group 512 and 256 for IgG and IgM, respectively. Serum was tested by real-time PCR by using a probe that enabled screening for spotted fever and a probe specific for *R. felis*; results were negative. A Western blot with cross-adsorption (*2*) showed *R. felis* as the causative agent (Figure). At a follow-up visit 3 months later, the patient had no signs or symptoms.**

**Rickettsiae were first described in the cat flea (*Ctenocephalides felis*) in 1918 and tentatively named *R. ctenocephali*. However, this work was overlooked until 1990, when an ELB agent was found in *C. felis* fleas by electron microscopy (*3*); the agent was demonstrated to be a *Rickettsia*-like organism. Results of subsequent studies were controversial because of suspected contamination of cultures. The species *R. felis* was formally validated by molecular criteria in 2001, and the reference strain was isolated in 2002 (*4*). *R. felis* has been demonstrated to belong to the SFG (*5*).**

***R. felis* is distributed worldwide (Technical Appendix), although it has not been found in the northern, coldest regions. The vectors described include fleas**, ticks, and mites; however, the only currently recognized vector is the *C. felis* flea (*6*). The reported hosts for these vectors are mainly cats, dogs, and rodents. *R. felis* is the only SFG species that is transmitted by fleas. Studies have confirmed that *R. felis* in *C. felis* flea populations is mostly maintained by transstadial and transovarial transmission (*7*). Levels of *R. felis* infestation in *C. felis* fleas are variable, and the specific mechanisms of maintenance within each flea remain unknown. Prevalence is increased by fleas feeding on mammalian hosts infected with *R. felis*. Nevertheless, the precise relationship between the vector and the host remains unknown, and the mechanisms of rickettsial replication have not yet been examined (*7*).

We searched PubMed and found reports (case reports and seroprevalence studies) of 68 *R. felis* infections. Cases have been reported in the Americas, Asia, Tunisia, and Europe (Technical Appendix). Such clinical cases rarely occur in warm countries, unlike the worldwide distribution of the bacteria, mentioned above. Reports of human infection with *R. felis* are rare, but the organism is frequently isolated from fleas.

We summarized the available clinical findings for 34 persons infected with *R. felis*: 32 had fever; 24, cutaneous rash (mostly maculopapular); 4, cutaneous eschar; 5, neurologic signs; 7, digestive symptoms; 3, cough without pneumonia; and 2, pneumonia. Clinical findings for *R. felis* are often confused with those found for patients with murine typhus or other febrile illnesses, and they appear to be more complex and more severe than initially thought.

***R. felis* infections can be diagnosed by serologic testing (*1*), molecular analysis, or a combination of each. Several molecular methods for detection of *R. felis* have focused on the presence of several genes, but real-time PCR assays are becoming increasingly useful (*8*). Serologic profiles for *R. felis* infections differ; cross-reactions with SFG rickettsiae as well as with SFG rickettsiae and *R. typhi* have been observed, but a lack of cross-reactions has also been observed (*9*). It has been noted (*10*) that when cross-reactions were observed between *R. felis* and both *R. conorii* and *R. typhi*, the infection was probably related to *R. felis*. When cross-reactions were observed between *R. felis* and only *R. typhi*, the causative agent was most probably of the typhus group.**

***R. felis* infections occur globally and are linked to the worldwide distribution of vectors, but the occurrence is relatively rare when compared with the high frequency of *R. felis* infections related to flea infestation. Human infections remain poorly characterized and are apparently underappreciated, possibly because of the lack of specific signs and symptoms. Further characterization of the interactions between *R. felis* and fleas could elucidate the particular epidemiology and pathology of flea-borne spotted fever.**

**Figure:**
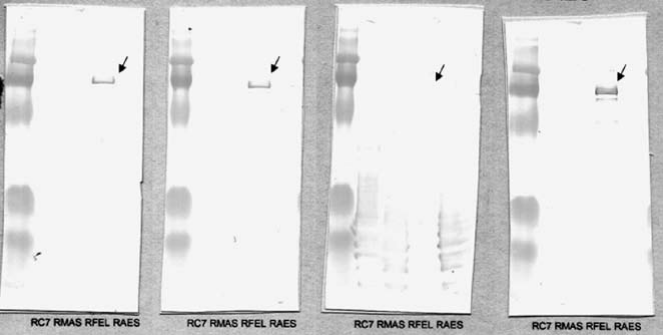
Western blot after cross-adsorption with (left to right) *Rickettsia conorii*, *R. massiliae*, *R. felis*, and *R. aeschlimannii*. When cross-adsorption is performed with *R. felis*, the specific antigen-corresponding line disappears, which indicates *R. felis* as the causative microorganism.

## Supplementary Material

Technical Appendix Rickettsia felis Infection in Man, France
