# Supplementary material for: Rickettsia felis Infection in Man, France
Source: Emerg Infect Dis. 2009 Jul;15(7):1126–7. doi: 10.3201/eid1507.090029 (PMC2744249; doi:10.3201/eid1507.090029)
Supplement: Technical Appendix — Rickettsia felis Infection in Man, France [file 09-0029-Techapp.pdf]

Rickettsia felis Infection in Man, France

Technical Appendix

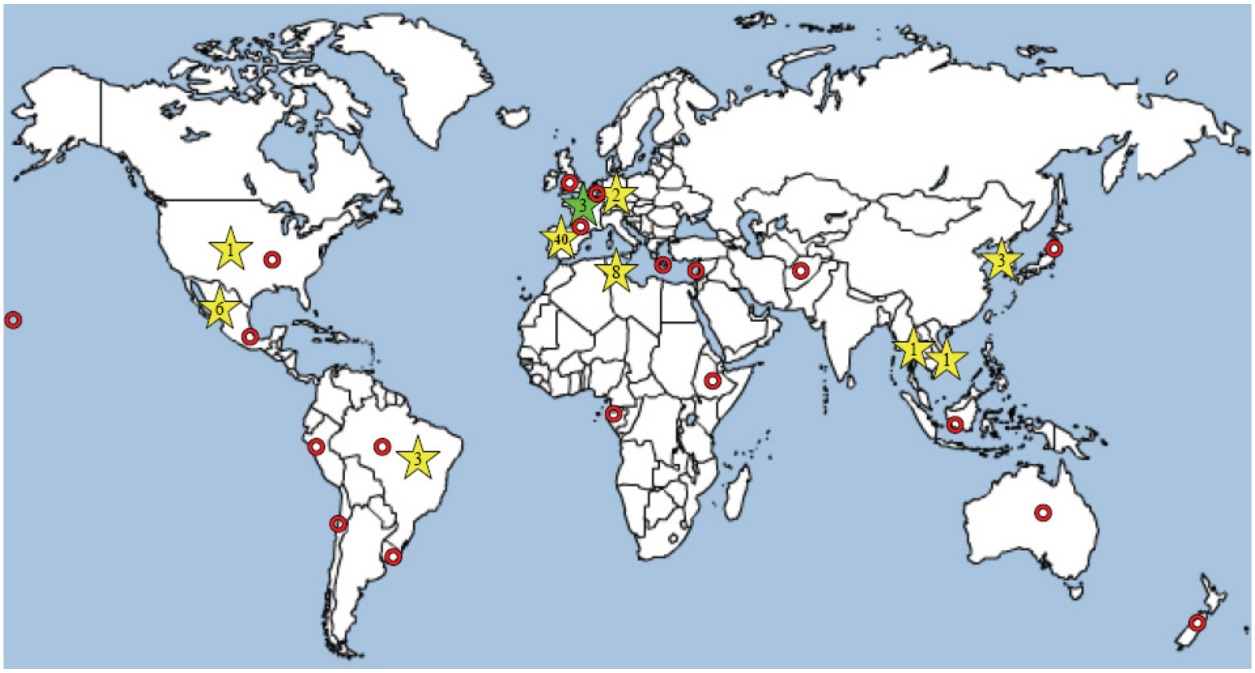

Figure. Distribution of clinical findings of *Rickettsia felis* infections and of reported potential vectors infected with *R. felis*. The numbers in the stars indicate the number of clinical cases (green star indicates case reported in this article), and the circles indicate locations of potential infected vectors per country. References are as follows: Texas (*1*), Mexico (*2–4*), Brazil (*3,5*), South Korea (*6*), Laos (*7*), Thailand (*8*), Tunisia (*9*), Germany (*10*), Spain (*11–14*), and France (5).

References

1. La Scola B, Meconi S, Fenollar F, Rolain JM, Roux V, Raoult D. Emended description of *Rickettsia felis* (Bouyer et al. 2001), a temperature-dependent cultured bacterium. Int J Syst Evol Microbiol. 2002;52:2035–41. Medline DOI: 10.1099/ijs.0.02070-0

2. Zavala-Velázquez JE, Ruiz-Sosa JA, Sánchez-Elias RA, Becerra-Carmona G, Walker DH. *Rickettsia felis* rickettsiosis in Yucatán. Lancet. 2000;356:1079–80. Medline DOI: 10.1016/S0140-6736(00)02735-5

3. Galvão MA, Zavala-Velazquez JE, Zavala-Castro JE, Mafra CL, Calic SB, Walker DH. *Rickettsia felis* in the Americas. Ann N Y Acad Sci. 2006;1078:156–8. Medline DOI: 10.1196/annals.1374.027

4. Zavala-Velazquez J, Laviada-Molina H, Zavala-Castro J, Pérez-Osorio C, Becerra-Carmona G, Ruiz-Sosa JA, et al. *Rickettsia felis*, the agent of an emerging infectious disease: report of a new case in Mexico. Arch Med Res. 2006;37:419–22. Medline DOI: 10.1016/j.arcmed.2005.08.003

5. Raoult D, La Scola B, Enea M, Fournier PE, Roux V, Fenollar F, et al. A flea-associated *Rickettsia* pathogenic for humans. Emerg Infect Dis. 2001;7:73–81. Medline

6. Choi YJ, Jang WJ, Ryu JS, Lee SH, Park KH, Paik HS, et al. Spotted fever group and typhus group rickettsioses in humans, South Korea. Emerg Infect Dis. 2005;11:237–44. Medline

7. Phongmany S, Rolain JM, Phetsouvanh R, Blacksell SD, Soukkhaseum V, Rasachack B, et al. Rickettsial infections and fever, Vientiane, Laos. Emerg Infect Dis. 2006;12:256–62. Medline

8. Parola P, Miller RS, McDaniel P, Telford SR III, Rolain JM, Wongsrichanalai C, et al. Emerging rickettsioses of the Thai-Myanmar border. Emerg Infect Dis. 2003;9:592–5. Medline

9. Znazen A, Rolain JM, Hammami A, Jemaa MB, Raoult D. *Rickettsia felis* infection, Tunisia. Emerg Infect Dis. 2006;12:138–40. Medline

10. Richter J, Fournier PE, Petridou J, Häussinger D, Raoult D. *Rickettsia felis* infection acquired in Europe and documented by polymerase chain reaction. Emerg Infect Dis. 2002;8:207–8. Medline

11. Bernabeu-Wittel M, del Toro MD, Nogueras MM, Muniain MA, Cardeñosa N, Márquez FJ, et al. Seroepidemiological study of *Rickettsia felis, Rickettsia typhi, and Rickettsia conorii* infection among the population of southern Spain. Eur J Clin Microbiol Infect Dis. 2006;25:375–81. Medline DOI: 10.1007/s10096-006-0147-6

12. Nogueras MM, Cardeñosa N, Sanfeliu I, Muñoz T, Font B, Segura F. Serological evidence of infection with *Rickettsia typhi* and *Rickettsia felis* among the human population of Catalonia, in the northeast of Spain. Am J Trop Med Hyg. 2006;74:123–6. Medline

13. Oteo JA, Portillo A, Santibáñez S, Blanco JR, Pérez-Martínez L, Ibarra V. Cluster of cases of human *Rickettsia felis* infection from southern Europe (Spain) diagnosed by PCR. J Clin Microbiol. 2006;44:2669–71. Medline DOI: 10.1128/JCM.00366-06

14. Pérez-Arellano JL, Fenollar F, Angel-Moreno A, Bolaños M, Hernández M, Santana E, et al. Human *Rickettsia felis* infection, Canary Islands, Spain. Emerg Infect Dis. 2005;11:1961–4. Medline
